# Self evaluation of communication experiences after laryngeal cancer – A longitudinal questionnaire study in patients with laryngeal cancer

**DOI:** 10.1186/1471-2407-8-80

**Published:** 2008-03-27

**Authors:** Mia Johansson, Anna Rydén, Caterina Finizia

**Affiliations:** 1 Department of Otolaryngology, Sahlgrenska University Hospital, SE 413 45 Göteborg, Sweden; 2 Health Care Research Unit, Sahlgrenska University Hospital, SE 413 45 Göteborg, Sweden; 3 Department of Otolaryngology, Sahlgrenska University Hospital Mölndal, SE 431 80 Mölndal Sweden

## Abstract

**Background:**

Aim of this longitudinal study was to investigate the sensitivity to change of the Swedish Self Evaluation of Communication Experiences after Laryngeal Cancer questionnaire (the S-SECEL), addressing communication dysfunction in patients treated for laryngeal cancer. Previous studies have highlighted the need for more specific questionnaires for this purpose.

**Methods:**

100 patients with Tis-T4 laryngeal cancer were included prior to treatment onset. Patients answered four questionnaires at six occasions during one year; the S-SECEL, the European Organisation for Research and Treatment of Cancer (EORTC) Core Quality of Life Core Questionnaire (QLQ-C30) supplemented by the Head and Neck cancer module (QLQ-H&N35) and the Hospital Anxiety and Depression (HAD) scale. In addition, performance status was assessed. Differences within groups were tested with the Wilcoxon paired signed ranks test and between-group analyses were carried out using the Mann-Whitney *U *test. Magnitude of group differences was analyzed by means of effect sizes.

**Results:**

The S-SECEL was well accepted with a response rate of 76%. Communication dysfunction increased at 1 month, followed by a continuous decrease throughout the year. Changes were statistically significant at most measurement, demonstrating the sensitivity of the S-SECEL to changes in communication over time. The S-SECEL and the EORTC QLQ-C30 with the QLQ-H&N35 demonstrated similar results; however the S-SECEL was more sensitive regarding communication dysfunction. The largest changes were found in the most diagnose specific items concerning voice and speech.

**Conclusion:**

The S-SECEL was investigated in the largest Scandinavian longitudinal study concerning health-related quality of life (HRQL) in laryngeal cancer patients. The questionnaire was responsive to change and showed convergent results when compared to established HRQL questionnaires. Our findings also indicate that the S-SECEL could be a more suitable instrument than the EORTC QLQ-C30 with QLQ-H&N35 when measuring communication experiences in patients with laryngeal cancer; it is more sensitive, shorter and can be used on an individual basis. As a routine screening instrument the S-SECEL could be a valuable tool for identifying patients at risk for psychosocial problems and to help plan rehabilitation. It is therefore recommended for clinical use in evaluation of communication dysfunction for all patients with laryngeal cancer irrespective of treatment.

## Background

Patients with head and neck cancer not only have to deal with the impact of a life-threatening disease, but also with the effect of treatment on physical, psychological, and social functioning. Studies have indicated that as many as one third of cancer patients suffer from psychiatric morbidity [1-3]. Regarding head and neck cancer the last decade has seen a growing interest regarding the health-related quality of life (HRQL) for these patients, and functional status have been recognized as important outcome variables in the evaluation of head and neck cancer treatment [4-6]. A number of reliable and valid HRQL questionnaires have been developed, e.g. the Functional Assessment of Cancer Therapy-General and the Head & Neck module (FACT-G & FACT-H&N) [7], the Performance Status Scale for Head and Neck Cancer Patients (PSS-HN) [8,9], the EORTC Core Quality of Life Questionnaire and the Head & Neck module (EORTC QLQ-C30 & EORTC QLQ-H&N35) [10,11], and these questionnaires have resulted in a better understanding of the impact of treatment in head and neck oncology. However, they include only few questions addressing voice and communication dysfunction, issues of particular importance to laryngeal cancer patients [12,13].

Therefore, a short but comprehensive self-report instrument measuring perceived adjustment to communication experiences in laryngeal cancer patients treated with laryngectomy, the Self Evaluation of Communication Experiences after Laryngectomy (SECEL), was developed and psychometrically evaluated in the United States [14]. The SECEL has been used as a screening tool to develop recommendations for intensive counselling, and for evaluating the effects of voice therapy and rehabilitation on the patients' daily living activities. For identifying the patients in need of further rehabilitation and in-depth counselling, the original authors have recommended a specific cut off value. [14,15]. The SECEL has been adapted to Swedish conditions and revised for use in laryngeal cancer patients receiving different treatment modalities [16]. The results lent support to the S-SECEL as a potentially useful outcome measure in patients with laryngeal cancer. It was also tested for sensitivity of change on a small group of patients (n = 26), where results after one year demonstrated a decrease in communication dysfunction [17].

The primary aim of the present study investigated whether the S-SECEL was sensitive to changes in communication and psychosocial dysfunction over time in a large cohort of laryngeal cancer patients. Secondary aim was to evaluate longitudinal score changes in relation to established and validated HRQL instruments.

## Methods

### Patients

All patients with newly diagnosed or relapsing laryngeal cancer in the western part of Sweden are continuously admitted to a weekly tumour conference at Sahlgrenska University Hospital, where the appropriate cancer treatment for each patient is discussed. During the inclusion period, all patients with laryngeal cancer were asked to participate in the current study were excluded from the study, as well as patients with some other concurrent cancer diagnosis and patients participating in other studies.

During the study period 210 patients with laryngeal cancer were admitted to the tumour conference, 100 were included in the study while 110 patients were excluded. Reasons for exclusion were poor general health (WHO performance status 3–4) or dementia diagnosed by a psychiatrist or GP (56), participation in other studies (19), poor knowledge of Swedish (10), family reasons (3), other concurrent cancer disease (3), and unknown reasons (2). Another 17 patients declined participation.

### Design

Data used in this prospective longitudinal study was derived from a battery of Patient Related Outcome (PRO) questionnaires. The study was ongoing between 1998 and 2005 with a discontinuation for two years. Results from repeated measures were compared for three forms; the S-SECEL, the HAD scale and the EORTC QLQ-C30 with QLQ-H&N35.

Participants answered questionnaires regarding HRQL and voice and speech on six occasions during a follow-up time of one year. Before start of treatment (baseline) questionnaires were distributed to patients at the tumour conference and mailed-back. A mail-out/mail-back procedure was used for follow-up assessments at 1, 2, 3, 6 and 12 months after start of treatment. Patients who had not returned their questionnaires within 2–3 weeks were reminded once by mail. Patients were further followed up with a visit to the outpatient clinic 12 months after treatment start, with recording of received treatment and evaluation of performance status and residual tumour.

At the conference, diagnosis according to TNM (UICC) and ICD and histopathology was recorded. Performance status was rated according to the WHO performance scale. For clinical purposes, the patients were asked about previous and present diseases, present symptoms, weight loss and smoking habits. Socio-demographic data such as family situation, education level, occupation, and smoking habits were also recorded.

### Treatment

All patients had received or were receiving radiotherapy as part of their treatment. The majority of the patients with T0-T1 disease received conventionally fractionated radiation therapy, a few received hyper fractionated radiation therapy. Patients with T2-T4 disease received either hyper fractionated radiotherapy or conventionally fractionated, in a majority the regional nodes were also irradiated as well. Chemotherapy was given to 9 patients with stage III-IV tumours. One patient was laryngectomised before inclusion in the study, 2 patients were treated with primary laryngectomy and 4 patients were treated with laryngectomy as salvage surgery during the study year.

### Questionnaires

#### S-SECEL

The original Self Evaluation of Communication Experiences after Laryngectomy (SECEL) was developed to assess communication dysfunction in patients with laryngectomies and has demonstrated satisfactory psychometric properties [14]. The Swedish version (S-SECEL, App) was adapted for use in patients who receive different treatments for laryngeal cancer. Two items in the original SECEL, specifically addressing experiences after laryngectomy, were re-worded in the S-SECEL. Otherwise the S-SECEL is congruent with the original SECEL in both its format and content. The S-SECEL has proved reliable and shown both convergent and discriminant validity and satisfactory internal consistency [16,17].

The questionnaire consists of 35 items addressing communication experiences and dysfunction (App I). 34 of the items are aggregated into three subscales. The first subscale, General (5 items), describes general attitudes about being relaxed or calm and acknowledgement of the sickness and treatment. Examples of questions are "Do you think that your speech improves with practice?" or "Would you describe yourself as outgoing and talkative?" The second subscale, Environmental (14 items), focuses on how the patient experiences his/her voice in different environments. Questions are for example; "Do you have trouble speaking in a large room?" and "Do you have difficulty yelling or calling out to people?" The third subscale, Attitudinal (15 items), describes attitudes about speech, feelings about self-perceptions and perceptions of others, for example "Do you avoid speaking because of your voice?" and "Do you feel that people get annoyed with you because of your voice?" Each item is rated on a 4-point categorical scale ranging from 0 (never) to 3 (always), and addressing the last 30 days. Scoring of subscales and a total scale is carried out by simple addition. Thus, the summary scale scores range from 0–15 for General, 0–42 for Environmental, 0–45 for Attitudinal and 0–102 for Total, respectively. A higher score indicates greater perceived communication dysfunction. The 35th item "Do you talk the same amount now as before your laryngeal cancer" has three response categories, Yes/More/Less, and is not included in the scoring system.

#### EORTC QLQ-C30 and QLQ-H&N35

The EORTC Study Group on Quality of Life has developed a modular measurement system for evaluating quality of life in cancer patients participating in clinical trials [18]. A 30-item core questionnaire, the EORTC QLQ-C30, assesses the physical and psychosocial functioning and symptom experiences of cancer patients in general [10]. To address additional symptoms associated specifically with head and neck cancer and its treatment, a complementary 35-item module can be used, the QLQ-H&N35 [19,20]. When tested in large, cross-cultural samples of patients with cancer, both the core questionnaire [10] and the head and neck cancer-specific module [20] have demonstrated satisfactory to excellent reliability and validity. Of particular importance is the ability of these questionnaires to distinguish between patient groups differing in clinical status and to detect changes in patients' clinical status over time.

Calculated scale scores range from 0–100. On the Functioning scales and Global quality of life scale a score of 100 corresponds to maximum functioning, whereas on the Symptom scales and items a score of 100 means worst possible symptoms [21]. A change in score over time of > 10 points should be interpreted as clinically significant [22].

### Hospital Anxiety and Depression (HAD) scale

The HAD scale is a tool detecting mood disorders in somatically ill patients [23] and has frequently been used in cancer studies, for example lung cancer [24] and head and neck cancer [25,26]. The 2-factor structure has been confirmed in many studies [27,28]. The Swedish version has been documented in several studies [29]. HAD consists of 14 items on a four-point response scale ranging from 0–3. The summary scale scores for anxiety (7 items) and depression (7 items) thus range from 0–21. Each person is also grouped according to a clinically tested classification of psychiatric morbidity. A scale score < 8 is in the normal range, a score 8–10 indicates a possible and a score > 10 indicates a probable mood disorder.

### Statistical methods

The statistical software SPSS 14.0 for Windows was used in the statistical analyses.

Descriptive statistics with 95% confidence interval (CI) were calculated according to standard procedures. Differences within groups were tested with the Wilcoxon paired signed ranks test and between-group analyses were carried out using the Mann-Whitney *U *test. Level of significance was set at 5% throughout. Clinical significance has also been calculated for EORTC, i.e. a score difference of >10 points. Magnitude of group differences was further analyzed by means of effect sizes (ES). ES of within-group change was calculated as mean change between assessments divided by the standard deviation of change [30]. ES were judged against standard criteria proposed by Cohen: trivial (0 to < 0.2), small (0.2 to < 0.5), moderate (0.5 to < 0.8) and large (≥ 0.8) [31]. This method supplements usual significance testing and provides standardized effect levels regardless of sample size and scaling properties.

### Ethical aspects

The study was conducted in accordance with the Declaration of Helsinki and was approved by the ethical committee at Sahlgrenska University Hospital, Göteborg, Sweden.

## Results

Socio-demographic characteristics of included and excluded patients are presented in Table 1. Significant differences between included and excluded patients were found regarding tumour site, stage of disease and WHO index. The excluded patients more often had a supraglottic tumour, advanced disease and worse performance status according to WHO-Index. No significant differences were found between the included and excluded patients concerning gender, age, or concurrent diseases.

**Table 1:** Clinical characteristics of included and excluded patients

	Included (n = 100)	Excluded (n = 110)	*p*-value^†^
Age, median years (range)	67 (27–92)	71 (44–87)	ns
Sex			ns
Female	17 (17 %)	22 (20 %)	
Male	83 (83 %)	88 (80 %)	
*Tumour site*			
Glottic	72 (72 %)	61 (55 %)	0.0188
Supraglottic	20 (20 %)	37 (34 %)	0.0382
Subglottic	4 (4 %)	3 (3 %)	ns
Transglottic	4 (4 %)	9 (8 %)	ns
*Stage*			
0	3 (3 %)	2 (2 %)	
I	57 (57 %)	43 (39 %)	
II	22 (22 %)	24 (22 %)	
III	9 (9 %)	17 (15 %)	
IV	9 (9 %)	24 (22 %)	0.0010
*Performance status WHO-Index*			
0	77 (77 %)	62 (59 %)	
1	18 (18 %)	25 (24 %)	
2	4 (4 %)	13 (12 %)	
3	1 (1 %)	4 (4 %)	
4		1 (1 %)	0.0016
Married/Cohabitant	70 (70 %)	62 (56 %)	ns
Smokers	50 (50 %)	70 (64 %)	ns
Loss of weight	21 (21 %)	35 (32 %)	ns
Residual disease	2 (2 %)	2 (2 %)	ns
Cardiovascular disease	45 (45 %)	38 (35 %)	ns
Other malignancy	8 (8 %)	11 (10 %)	ns

^†^p-value significant at ≤ 0.05, ns = not significant

The included patients were classified as N0M0, except one patient classified as N2M0 and two classified as N2M1. In the excluded group 10 patients were classified as N1M0, six as N2M0, one as N3M0 and one patient as N2M1.

The questionnaire response rate was 100% at baseline, 95% at one month, 86% at two months, 81% at three months, 75% at six months and 71% at one year. Of the 71 patients completing the study five were laryngectomised while 66 (93%) had preserved larynx. At the end of the study year five patients still had active disease or were deceased. Thus, the cumulative response rate of patients answering all six questionnaires over one year was 76 %, and the item response rate was 99.5%, i.e., only 0.5% of the questions were left unanswered The 29 drop-out patients missing at follow-up did not differ from the participants completing the study regarding gender, age, civil status or educational level but significantly more were smokers and had a supraglottic localisation (data not shown).

### Questionnaires

#### S-SECEL

No significant differences in S-SECEL baseline scores were found between the 29 drop-out patients and the 71 patients completing the study

**Figure 1:**
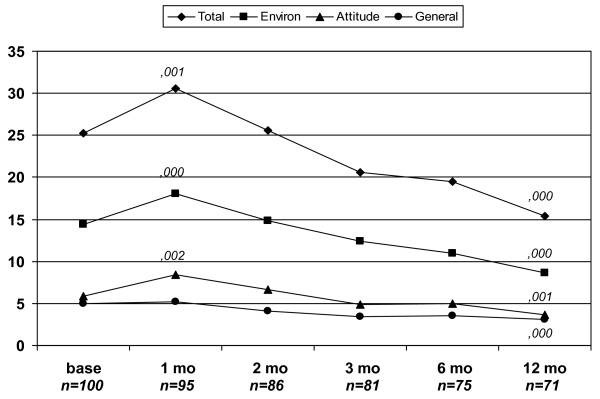
**Mean S-SECEL total and subscale scores**. Shows the mean values over time during the study year for S-SECEL total score and subscales. All patients who answered the questionnaires at each measurement point were included in the figure, while the p-values refer to Wilcoxon signed rank test in patients with both baseline and follow-up assessments. S-SECEL = Swedish Self Evaluation of Communication Experiences after Laryngeal Cancer. env = environmental subscale, gen = general subscale, att = attitudinal subscale, tot = total score.

**Figure 2:**
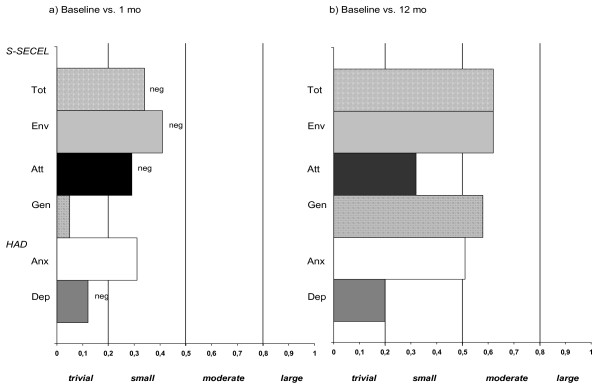
**Changes (effects sizes) in S-SECEL and HAD**. Changes (effects sizes) in S-SECEL and HAD between (*a*) baseline and 1 month follow-up and (*b*) baseline and 12 month follow-up; neg = negative change, i.e. increased dysfunction and anxiety/depression.

The mean S-SECEL total and subscale scores at the different measurement points are shown in Table 2 and Figure 1. As can be seen, patients report an increase in speech dysfunction between baseline and one month on all subscales. Changes are statistically significant for the Total, Attitudinal, and Environmental scales but these changes are, in ES terms, small (Fig 2). From 2 months throughout the year there is a continuous decrease in perceived dysfunction on all subscales and the Total scale. Changes between baseline and 12 months were statistically significant for all subscales. ES differences are regarded as moderate for the Total, Environmental and General scales but only small for the Attitudinal scale (Fig 2).

**Table 2:** Mean values *(CI) *for S-SECEL scores before treatment (baseline) and follow-up and p-values and effect sizes for changes at follow-up compared to baseline

	Base, n = 100	1 mo, n = 95	2 mo, n = 86	3 mo, n = 81	6 mo, n = 75	12 mo, n = 71
*S-SECEL*						
Total*	25.2 *(22.5–28.0)*	30.6 *(27.3–33.8)*	25.6 *(21.6–29.6)*	20.6 *(17.2–24.1)*	19.5 *(15.3–23.7)*	15.4 *(11.9–18.9)*
*p*^†^/ES^‡^		0.00/0.34	ns/0.03	0.02/0.24	0.00/0.32	0.00/0.62
Environment*	14.4 *(12.8–16.0)*	18.1 *(16.2–19.9)*	14.9 *(12.8–17.0)*	12.4 *(10.4–14.4)*	11.0 *(8.9–13.1)*	8.7 *(6.8–10.5)*
*p*^†^/ES^‡^		0.00/0.41	ns/0.04	ns/0.20	0.00/0.35	0.00/0.62
Attitudinal*	5.9 *(4.7–7.1)*	8.4 *(6.6–10.2)*	6.7 *(5.0–8.4)*	4.9 *(3.6–6.1)*	4.9 *(3.0–6.8)*	3.7 *(2.1–5.3)*
*p*^†^/ES^‡^		0.00/0.29	ns/0.09	ns/0.10	0.50/0.11	0.00/0.32
General*	5.0 *(4.5–5.4)*	5.2 *(4.1–6.2)*	4.1 *(3.5–4.6)*	3.4 *(2.9–3.9)*	3.6 *(3.0–4.1)*	3.1 *(2.5–3.7)*
*p*^†^/ES^‡^		ns/0.05	0.05/0.25	0.00/0.55	0.00/0.53	0.00/0.58

^†^p-value significant at ≤ 0.05, ns = non significant

*Min-max subscales: 0–15 (general), 0–42 (environmental), 0–45 (attitudinal), and 0–102 (total)

^‡^Effect size (ES) of change was calculated as mean change from baseline divided by the standard deviation of change. Criteria: trivial 0 to < 0.20; small 0.20 to < 0.50; moderate 0.50 to < 0.80; and large ≥ 0.80

Two items explained most of the difference in the Environmental scale: 'difficulty yelling or calling out to people' and 'trouble speaking in large groups' (Mdiff = 0.60/0.54). These questions also displayed the largest dysfunction before treatment. For the General scale one item differed more than the others: 'does your speech improve with practice' (Mdiff = 0.89) while the smallest change was noted for 'are you a calm and quiet person'. Items in the Attitudinal scale scored low both before and after treatment but the greatest change was found for 'is your private or social life limited' (Mdiff = 0.37)

#### EORTC QLQ-C30 and QLQ-H&N35

Data for the EORTC QLQ-C30 and QLQ-H&N35 scales and items are shown in Table 3 and 4. One month after treatment start, statistically significant deterioration was seen in the Role, Social, Emotional and Global QLQ functioning scales, this corresponded to a clinical deterioration only for Role functioning. Except for Social functioning, at the 12 month follow-up clinical and/or statistical significant improvements, compared to baseline, were reported for all of these scales. For the symptom scales, an increase in mean scores was seen on all measures at one month, indicating deterioration. At the last follow-up, however, there were no significant changes compared to baseline.

**Table 3:** Mean values *(CI) *for EORTC QLQ-C30 scores before treatment (baseline) and follow-up and p-values and effect sizes for changes at follow-up compared to baseline

	Base, n = 100	1 mo, n = 95	2 mo, n = 86	3 mo, n = 81	6 mo, n = 75	12 mo, n = 71
EORTC QLQ-C30						
Functional scales*						
Physical	85.1 *(81.5–88.7)*	81.7 *(78.1–85.3)*	81.4 *(77.4–85.4)*	84.2 *(80.5–87.9)*	85.6 *(81.7–89.5)*	88.5 *(85.2–91.9)*
p^†^/ES^‡^		.006/0.32	.001/0.35	.038/0.25	ns/0.18	ns/0.04
Role	77.8 *(71.8–83.8)*	65.4 ^§ ^*(58.7–72.2)*	74.1 *(67.3–81.0)*	80.7 *(74.7–86.6)*	85.4 *(79.7–91.0)*	89.2^§ ^1 *(84.5–93.9)*
p^†^/ES^‡^		0.000/0.41	0.011/0.25	ns/0.08	ns/0.06	ns/0.19
Social	86.9 *(82.6–91.2)*	80.5 *(75.7–85.4)*	83.5 *(78.3–88.8)*	87.2 *(82.7–91.8)*	88.0 *(82.5–93.5)*	90.1 *(85.5–94.8)*
p^†^/ES^‡^		.005/0.26	ns/0.16	ns/0.09	ns/0.07	ns/0.07
Emotional	74.4 *(69.7–79.1)*	76.5 *(71.9–81.1)*	81.7 *(77.7–85.6)*	84.6^§ ^*(80.6–88.6)*	83.4 *(78.0–88.8)*	89.5^§ ^*(85.7–93.2)*
p^†^/ES^‡^		ns/0.09	.042/0.26	.002/0.37	.009/0.33	.000/0.61
Cognitive	83.5 *(79.5–87.5)*	84.0 *(80.3–87.7)*	87.8 *(84.3–91.3)*	88.1 *(84.4–91.7)*	87.6 *(83.7–91.5)*	88.3 *(84.8–91.7)*
p^†^/ES^‡^		ns/0.02	ns/0.15	ns/0.16	ns/0.12	ns/0.19
Global QLQ	68.4 *(64.1–72.6)*	63.5 *(59.1–67.9)*	69.2 *(64.7–73.7)*	72.7 *(68.4–77.1)*	75.1 *(69.9–80.3)*	77.9 *(73.0–82.9)*
p^†^/ES^‡^		.015/0.24	ns/0.06	ns/0.08	ns/0.13	.007/0.31
Symptom scales*						
Pain	17.7 *(12.7–22.7)*	35.4^§ ^*(29.8–41.1)*	23.5 *(18.0–28.9)*	17.7 *(12.7–22.7)*	14.9 *(9.2–20.6)*	8.7 *(4.7–12.7)*
p^†^/ES^‡^		.000/0.67	.002/0.35	ns/0.13	ns/0.04	ns/0.04
Fatigue	25.1 *(20.6–29.7)*	39.3^§ ^*(34.1–44.5)*	34.6 *(29.1–40.2)*	30.3 *(25.3–35.3)*	21.2 *(16.3–26.1)*	18.2 *(13.6–22.7)*
p^†^/ES^‡^		.000/0.63	.000/0.52	.000/0.41	ns/0.00	ns/0.16
Nausea/vomiting	5.2 *(2.9–7.4)*	14.2 *(9.7–18.8)*	12.0 *(8.0–16.0)*	7.0 *(3.2–10.8)*	2.7 *(1.0–4.3)*	1.4 *(0.3–2.5)*
p^†^/ES^‡^		.000/0.40	.000/0.42	ns/0.19	ns/0.05	ns/0.19

*Min-max: 0–100

^§ ^Indicates clinical significant change measured from baseline, i.e. a change of > 10 points

^† ^p-value significant at ≤ 0.05, ns = not significant.

^‡ ^Effect size (ES) of change was calculated as mean change from baseline, divided by the standard deviation of change. Criteria: trivial 0 to < 0.20; small 0.20 to < 0.50; moderate 0.50 to < 0.80; and large ≥ 0.80

**Table 4:** Mean values *(CI) *for QLQ-H&N35 scores before treatment (baseline) and follow-up and p-values and effect sizes for changes at follow-up compared to baseline

	Base, n = 100	1 mo, n = 95	2 mo, n = 86	3 mo, n = 81	6 mo, n = 75	12 mo, n = 71
EORTC QLQ-H&N35						
Scales*						
Pain	9.3 *(6.4–12.2)*	28.5^§ ^*(23.9–33.0)*	17.0 *(13.2–20.9)*	13.0 *(9.2–16.7)*	12.3 *(7.8–16.7)*	6.8 *(3.2–10.4)*
p^†^/ES^‡^		.000/0.99	.000/0.55	.016/0.28	ns/0.21	ns/0.08
Swallowing	6.8 (4.2–9.5)	33.1^§ ^*(27.7–38.5)*	22.8^§ ^*(17.4–28.1)*	14.3 *(9.1–19.6)*	11.3 *(6.8–15.7)*	6.8 *(3.3–10.3)*
p^†^/ES^‡^		.000/0.96	.000/0.68	.000/0.39	.005/0.34	ns/0.15
Senses	5.2 *(2.4–7.9)*	23.0^§ ^*(17.3–28.6)*	19.2^§ ^*(13.1–25.3)*	13.2 *(8.6–17.8)*	9.6 *(5.0–14.2)*	7.8 *(3.6–11.9)*
p^†^/ES^‡^		.000/0.70	.000/0.54	.000/0.49	.005/0.34	.010/0.31
Speech	37.2 *(32.5–41.9)*	44.9 *(39.5–50.4)*	34.4 *(28.3–40.6)*	24.6^§ ^*(19.0–30.1)*	24.3^§ ^*(18.3–30.3)*	16.0^§ ^*(11.8–20.2)*
p^†^/ES^‡^		.013/0.25	ns/0.09	.001/0.40	.001/0.45	.000/0.84
Social eating	7.1 *(4.1–10.0)*	29.1^§ ^*(23.3–34.9)*	17.4^§ ^*(12.6–22.3)*	11.0 *(6.3–15.7)*	7.3 *(3.3–11.4)*	6.0 *(2.1–9.9)*
p^†^/ES^‡^		.000/0.75	.000/0.50	.014/0.28	ns/0.17	ns/0.09
Social contact	6.9 *(4.5–9.3)*	9.4 *(6.6–12.1)*	7.6 *(5.2–10.1)*	5.2 *(2.4–8.1)*	4.5 *(1.4–7.5)*	2.8 *(0.3–5.3)*
p^†^/ES^‡^		.049/0.18	ns/0.10	ns/0.05	ns/0.16	.003/0.36
Sexuality	33.0 *(25.4–40.6)*	36.6 *(28.9–44.3)*	37.2 *(28.9–45.5)*	34.9 *(27.1–46.6)*	27.1 *(19.4–34.9)*	27.0 *(19.2–34.7)*
p^†^/ES^‡^		ns/0.12	ns/0.12	ns/0.17	ns/0.05	ns/0.01
Single items*						
Dry mouth	22.7 *(16.7–28.7)*	39.30^§ ^*(32.5–46.1)*	34.1^§ ^*(27.1–41.1)*	34.6^§ ^*(27.3–41.9)*	34.7^§ ^*(27.3–42.1)*	30.1 *(23.4–36.7)*
p^†^/ES^‡^		.000/0.52	.000/0.44	.000/0.53	.000/0.54	.002/0.40
Sticky saliva	20.2 *(14.8–25.6)*	50.7^§ ^*(43.7–57.7)*	45.0^§ ^*(37.4–52.5)*	37.1^§ ^*(29.9–44.3)*	38.7^§ ^*(30.9–46.4)*	31.9^§ ^*(25.1–38.7)*
p^†^/ES^‡^		.000/0.91	.000/0.80	.000/0.68	.000/0.66	.000/0.55
Coughing	29.0 *(24.0–34.0)*	48.1^§ ^*(42.0–54.2)*	40.3^§ ^*(33.5–47.1)*	35.4 *(29.2–41.6)*	32.4 *(25.0–39.9)*	24.9 *(18.8–31.0)*
p^†^/ES^‡^		.000/0.63	.004/0.33	.026/0.26	ns/0.21	ns/0.02
Felt ill	17.0 *(12.4–21.6)*	28.4^§ ^*(22.4–34.4)*	19.0 *(13.7–24.3)*	16.1 *(11.1–21.1)*	10.2 *(6.2–14.2)*	8.5 *(4.5–12.4)*
p^†^/ES^‡^		.000/0.0.45	ns/0.17	ns/0.08	ns/0.20	.034/0.26

*Min-max: 0–100

^§ ^Indicates clinical significant change measured from baseline, i.e. a change of >10 points.

^†^p-value significant at ≤ 0.05, ns = not significant.

^‡^Effect size (ES) of change was calculated as mean change from baseline, divided by the standard deviation of change. Criteria: trivial 0 to < 0.20; small 0.20 to < 0.50; moderate 0.50 to < 0.80; and large ≥ 0.80

For the H&N35, an increase was seen in all scales, but Sexuality, as well as all single items at one month, and most of these were both clinically and statistically significant. At the 12 month follow-up an improvement compared to baseline was reported for two of the scales and one item. However, patients still reported more problems with Senses and Sticky saliva.

#### HAD

One month after treatment start, patients reported a small but significant decrease in mean anxiety scores compared to baseline (Table 5, Fig 2). At the 12 month follow-up mean values had further decreased, the effect now being moderate. There were no significant changes for depression. According to HAD classifications, anxiety was more prevalent than depression at baseline, 34% vs. 15%. At the one month and 12 month follow-up 21% vs. 11% of the patients had possible/probable anxiety disorder. Corresponding figures for possible/probable depression was 21% vs. 8%.

**Table 5:** Mean values *(CI) *for HAD scores before treatment (baseline) and follow-up and p-values and effect sizes for changes at follow-up compared to baseline

*HAD*	Base, n = 100	1 mo, n = 95	2 mo, n = 86	3 mo, n = 81	6 mo, n = 75	12 mo, n = 71
Anxiety*	5.4 *(4.5–6.3)*	4.1 *(3.2–5.0)*	3.1 *(2.4–3.8)*	4.5 *(2.8–4.3)*	3.4 *(2.5–4.4)*	3.2 *(2.2–4.1)*
*p*^†^/ES^‡^		0.00/0.31	0.00/0.50	0.00/0.37	0.00/0.41	0.00/0.51
Depression*	3.7 *(3.0–4.5)*	4.0 *(3.2–4.8)*	3.2 *(2.6–3.8)*	2.9 *(2.3–3.5)*	2.8 *(2.0–3.6)*	2.5 *(1.7–3.3)*
*p*^†^/ES^‡^		ns/0.12	ns/0.03	ns/0.08	ns/0.07	ns/0.20

*Min-max score 0–21

^†^p-value significant at ≤ 0.05, ns = not significant

^‡^Effect size (ES) of change was calculated as mean change from baseline, divided by the standard deviation of change. Criteria: trivial 0 to < 0.20; small 0.20 to < 0.50; moderate 0.50 to < 0.80; and large ≥ 0.80

## Discussion

Results from our study proved the S-SECEL to be sensitive to changes in communication and psychosocial dysfunction longitudinally. The response pattern over time, when compared to the EORTC QLQ-C30 with H&N35 and the HAD, lent further support to the construct validity of the S-SECEL. The response rate for the used set of questionnaires was high, 76% at the last follow-up, supporting the feasibility of assessment method in clinical settings.

When looking at the individual S-SECEL scales, at the 12-month follow-up the decrease in the General scale is moderate. However, it should be noted that patients report little dysfunction before as well as after treatment. The General scale displays a somewhat different pattern to the other subscales during the year. There is no decrease at one month but already at the two month follow-up a small but significant improvement is seen. This different pattern might be explained by items being somewhat disparate, covering questions about voice quality as well as personality, and therefore of differing relevance. The question "Do you think your speech improves with practice" changes the most over the study period and this is a highly relevant question since it detects the effect of voice rehabilitation measures. It should be noted, that there is no corresponding question in the EORTC. The question "Would you describe yourself as a calm and quiet person?" changes the least, probably due to this question rather measuring a trait of character then effects of illness or treatment.

Both the Environmental and the Attitudinal subscales show a small but significant increased dysfunction one month after start of treatment. This is reasonable since the patients still receive treatment and therefore are suffering from various side effects, also affecting the voice. At the following measuring points scores decrease, but the decrease is not significant until the 6 month follow-up and these results are in accordance with previous studies [6,17,32]. Although both being significant at the 12 month follow-up the scales differ concerning effects sizes. As the General scale, the effect in the Environmental scale is moderate but only small for the Attitudinal. This is probably explained by the fact that the General and Environmental subscales include more voice specific questions than the Attitudinal, where items are more related to mental distress and self-esteem.

When reviewing the results of the EORTC QLQ-C30 and the QLQ-H&N35, we find diverging results for the different methods of significance testing. In a number of scales, at several measurement points, there are statistical but no clinical significances and effect sizes are small or even trivial. This illustrates the additional value of not using p-values alone as measure of significance.

In the EORTC QLQ-C30, only two of the scales, Role and Emotional, show significant changes at 12 months. The questions that constitute these scales have their equivalence in the S-SECEL. The Role scale consists of two questions concerning how the disease affects patients' ability to function in daily activities, work and leisure. This area is more specifically covered in the subscale Environmental of the S-SECEL. The Emotional scale in the EORTC consists of four questions about feeling annoyed, depressed, tense or worried. Some of these symptoms are covered in the Attitudinal scale and there in relation to voice and speech. This is an important distinction since, for example, one can feel annoyed in a specific situation such as a conversation without feel annoyed in general.

The significant changes in C30 and H&N35, statistically as well as clinically, are mainly seen in items in the head and neck module. All single items show significant changes one month after start of treatment as well as four of the scales. As with the S-SECEL, these changes probably reflect treatment effects. At one year there is only one clinically significant improvement reported, the Speech scale. It is well known that the main problems for patients with laryngeal cancer are voice related. The Speech scale in the QLQ-H&N35, however, consists of only two questions directly addressing quality of voice and speech; "Difficulties speaking to other people?" and "Problems speaking on the phone?". The scales Senses and Social contact and the single item Dry mouth show statistical significance at 12 months, but the effect sizes were only small and not clinically significant. Müller et al [13] argue that the QLQ-H&N35 are not specific enough and stress that questionnaires should be more precise when used on patients with laryngeal cancer. Op de Coul et al [12] have also underlined the necessity to develop and use more specific additional questionnaires as an adjunct to the existing EORTC questionnaires when studying specific symptoms in laryngectomised patients. In the SECEL items are more detailed, e.g. questions about speaking to others specify if it concerns speaking in large or small groups or to an individual.

Baseline results of the HAD scale corresponds well to other comparable studies [16,33], both regarding Anxiety and Depression. The decrease in Anxiety reported at the one month follow-up may seem contradictory to the increased dysfunction according to the S-SECEL. However, this could be due to a feeling of relief having been diagnosed and receiving treatment. This improvement, however, was not found in the Emotional scale of the EORTC QLQ-C30. Singer et al [34] found that the HAD scale showed a higher rate of accuracy than the EORTC's Emotional scale when measuring psychiatric morbidity in laryngeal cancer patients, which might explain this discrepancy. At the 12 month follow-up mean values for Anxiety had further decreased, the improvement in mental health now also being reflected in the Emotional scale.

Mean values of depression was low at baseline and did not change, a finding in line with previous studies, e.g. [33]. However, the prevalence of patients with possible/probable depression increased after treatment start and this might be due to the increase in dysfunction following treatment. After one year, and terminated treatment, the prevalence was lower than at baseline. We have used the recommended cut off values for possible and probable Depression and Anxiety; however it has been discussed whether there is a need for different cut off values for different populations [34,35]. For example, Zöger et al used the HAD when screening for anxiety and depression in patients with tinnitus, and found optimal cut-off score for these patients to be ≥ 5 [36].

A shortcoming of our study is that the patients excluded from the study had a more advanced disease and lower performance status than those included. This might have lead to an underestimation of the prevalence of psychiatric morbidity and communication dysfunction. On the other hand, this is to our knowledge the largest Scandinavian longitudinal study made on HRQL and communication dysfunction in patients treated for laryngeal cancer.

## Conclusion

The S-SECEL has been investigated in the largest Scandinavian longitudinal study concerning HRQL in patients with laryngeal cancer. The questionnaire is sensitive to change over time and shows convergent results when compared to established HRQL questionnaires. Our findings also indicate that the S-SECEL could be a more suitable instrument than the EORTC QLQ-C30 with QLQ-H&N35 in patients with laryngeal cancer; it is more sensitive, it is shorter and can be used on an individual basis. As a routine screening instrument, with the use of a specific cut off value, the S-SECEL could be a valuable tool for identifying patients at risk for psychosocial problems, to help to plan rehabilitation. It is therefore recommended for clinical use in evaluation of communication dysfunction for all patients with laryngeal cancer irrespective of treatment.

## Competing interests

The author(s) declare that they have no competing interests.

## Authors' contributions

We hereby certify our personal contribution; CF was responsible for the design and planning of the study. AR carried out the statistical analysis. MJ performed the literature search/analysis. AR and MJ were responsible for the data interpretation. All authors participated in preparation of manuscript. All authors read and approved the final manuscript.

## Appendix

### General scale

1) feel relaxed and comfortable in social settings

2) describe yourself as calm and quiet

3) describe yourself as outgoing, talkative

4) admit having had laryngeal cancer

5) your speech improves with practice

### Environmental scale

1) limited in meetings due to speech

2) difficulty getting peoples' attention

3) difficulty yelling or calling out to people

4) people having difficulty to understand

5) repeat things to be understood

6) trouble speaking in large groups

7) trouble speaking in small groups

8) trouble speaking with one person

9) trouble speaking in different rooms

10) trouble speaking in loud and noisy places

11) trouble speaking on telephone

12) trouble speaking in car or bus

13) difficulty attending parties or social activities

14) use telephone less often

### Attitudinal scale

1) feel left out in group

2) limited private or social life

3) depressed due to speech

4) frustrated when not understood

5) different or peculiar

6) hesitate to meet new people

7) get left out of conversations

8) avoid speaking

9) people fill in words

10) people interrupt

11) people tell you they can't understand

12) people get annoyed

13) people avoid you

14) people speak differently to you

15) family and friends difficulty understanding your communication situation

Response alternatives: Always/Most of the time/Sometimes/Never

### Single item

1) Do you talk the same amount now as before your laryngeal cancer?

Response alternatives: Yes/More/Less

## Pre-publication history

The pre-publication history for this paper can be accessed here:


